# Severe Myxedema Coma With Neurological, Cardiac, and Renal Involvement: A Case Report

**DOI:** 10.7759/cureus.98391

**Published:** 2025-12-03

**Authors:** Luis Fernando Domínguez-Valdez, Amilcar Rivero-Rejón, Jaime Enrique Hernández Utrera

**Affiliations:** 1 Endocrinology, Diabetes and Metabolism, Hospital General de México “Dr. Eduardo Liceaga”, Mexico City, MEX; 2 Internal Medicine, Unidad Médica de Alta Especialidad (UMAE) Hospital de Especialidades “Dr. Antonio Fraga Mouret”, Centro Médico Nacional “La Raza”, Mexico City, MEX; 3 Endocrinology, Instituto Nacional de Ciencias Médicas y Nutrición “Salvador Zubirán”, Mexico City, MEX; 4 Endocrinology, Centro Médico Nacional “La Raza”, Mexico City, MEX

**Keywords:** acute kidney injury, endocrine emergency, multiorgan dysfunction, myxedema coma, severe hypothyroidism

## Abstract

Myxedema coma is the most severe manifestation of hypothyroidism, with high mortality despite treatment. It typically affects elderly women and is often triggered by infection, drug exposure, or environmental stressors. We report the case of a 66-year-old woman with no prior history of thyroid disease who presented with progressive neurological deterioration, hypothermia, bradycardia, hypotension, and multiorgan dysfunction. Laboratory evaluation confirmed severe hypothyroidism with elevated thyroid-stimulating hormone and undetectable free T4, alongside markedly elevated cardiac enzymes, global cardiomyopathy with pericardial effusion, and acute kidney injury. Management with high-dose enteral levothyroxine and intravenous hydrocortisone, in combination with supportive care, led to rapid improvement, extubation by day three, and full recovery of thyroid, cardiac, renal, and neurological function. This case underscores the importance of early recognition and timely intervention in patients with unexplained encephalopathy and systemic compromise, as prompt treatment can reverse even severe presentations of myxedema coma.

## Introduction

Myxedema coma is the most severe form of hypothyroidism, with an incidence of 0.22-1.08 cases per million annually. It occurs predominantly in elderly women. In iodine-sufficient regions, autoimmune thyroiditis, particularly Hashimoto’s thyroiditis, is the leading cause. Typical manifestations include altered mental status, hypothermia, hypotension, bradycardia, hyponatremia, and hypoventilation [1,2]. Precipitating factors such as infection, trauma, surgery, or drugs like lithium and amiodarone have been reported [3]. Mortality remains high, ranging from 25% to 60%, especially in patients with advanced age, persistent hypothermia, or need for mechanical ventilation [4]. Diagnosis relies on clinical suspicion and confirmation of severe thyroid hormone deficiency. Treatment consists of thyroid hormone replacement, empiric corticosteroids, and supportive care with ventilation and hemodynamic stabilization [2,5,6]. Uncommon complications include rhabdomyolysis, acute kidney injury, and cardiovascular involvement such as arrhythmias or coronary syndromes [6,7]. In this report, we present the case of a 66-year-old woman with neurological deterioration and multiorgan dysfunction as the initial manifestation of myxedema coma, showing remarkable recovery after early therapy. Written informed consent for publication of clinical details and accompanying images was obtained from the patient.

## Case presentation

**Figure 1:**
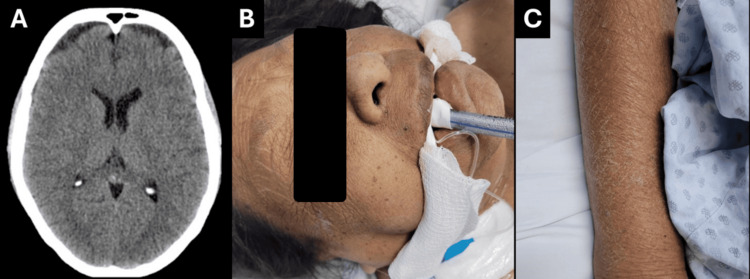
Clinical presentation of myxedema coma at admission. (A) Non-contrast brain CT showing no structural abnormalities. (B) The patient on arrival to the intensive care unit, showing characteristic generalized facial puffiness, macroglossia, dry coarse skin, and myxedematous features while under mechanical ventilation for hypoventilation and altered mental status. (C) Xerotic, thickened skin with scaling and rough texture on the forearm, consistent with severe hypothyroidism.

A 66-year-old woman with no significant past medical history or recent infections, medication changes, trauma, or vascular events presented to the emergency department with progressive neurological decline. She reported four weeks of fatigue and generalized muscle weakness, which culminated in acute somnolence on the day of admission. Brain CT revealed no structural abnormalities (Figure 1A); however, her neurological status deteriorated rapidly, with a Glasgow Coma Scale score of 7 (E:1, V:2, M:4) and oxygen desaturation requiring endotracheal intubation and mechanical ventilation. On arrival to the intensive care unit, she developed hypotension, hypothermia, bradycardia, and low cardiac output, necessitating vasopressor support (Figures 1B, 1C).

**Figure 2:**
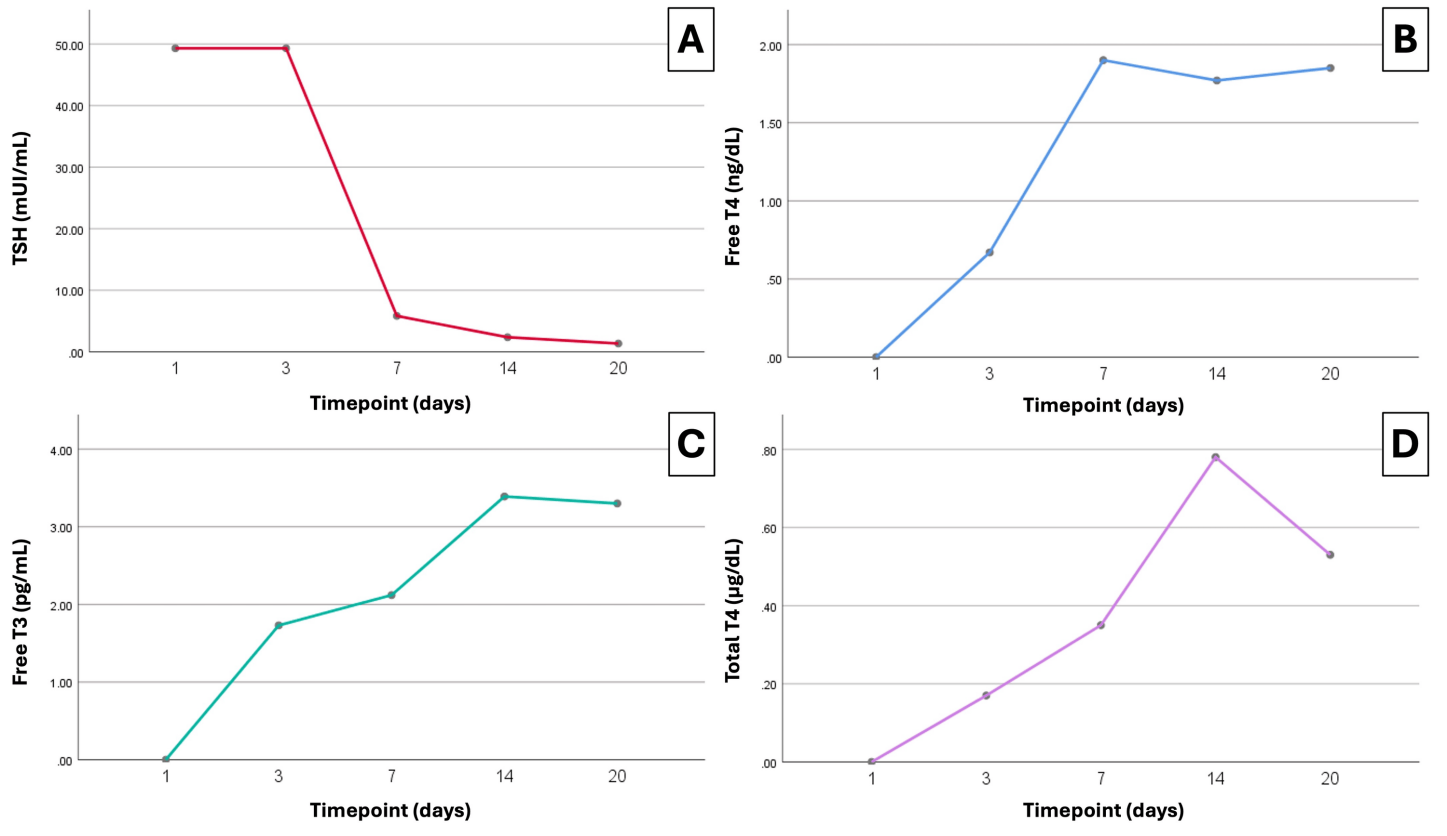
Serial thyroid function profile during hospitalization and follow-up. (A) Thyroid-stimulating hormone (TSH) shows persistently elevated levels at baseline and day three, followed by a steep decline by day seven and near-normalization by day 20. (B) Free thyroxine (T4) increases rapidly after initiation of high-dose enteral levothyroxine, reaching the reference range by day seven and stabilizing thereafter. (C) Free triiodothyronine (T3) demonstrates a progressive rise, with normalization achieved by day 14. (D) Total T4 rises steadily throughout treatment, peaking by week two.

Initial laboratory evaluation demonstrated severe hypothyroidism, with a thyroid-stimulating hormone (TSH) concentration of 49.3 mIU/mL and undetectable free thyroxine (free T4: 0.00 ng/dL) (Figure 2). The Popoveniuc score was 115, and the Chiong score was 8, consistent with a diagnosis of myxedema coma.

Following the confirmation of myxedema coma, treatment was initiated with intravenous hydrocortisone (200 mg bolus followed by 100 mg every eight hours for 48 hours, then tapered and discontinued once hemodynamic stability was achieved on day three) and high-dose enteral levothyroxine (400 µg daily via nasogastric tube for the first three days, followed by 150 µg daily). Levothyroxine therapy was continued throughout hospitalization until complete biochemical and clinical recovery. During early stabilization, mild hyponatremia and borderline hypoglycemia were corrected conservatively, and intensive care management included controlled ventilation, cautious fluid administration, vasopressor support, and passive rewarming to maintain hemodynamic stability.

Cardiac biomarkers were profoundly elevated at presentation; however, electrocardiography showed no evidence of acute coronary syndrome. Echocardiography demonstrated global hypokinesia with pericardial effusion, while renal involvement was evident with acute kidney injury and elevated urea levels. The patient’s clinical status improved rapidly, with discontinuation of vasopressors within 24 hours and successful extubation on day three. Serial laboratory monitoring revealed progressive normalization of thyroid function, recovery of renal parameters, and a marked decline in muscle and cardiac injury markers (Table 1).

**Table 1:** Evolution of cardiac, renal, and hematologic parameters during hospitalization. Serial laboratory values showing progressive improvement after initiation of enteral levothyroxine and intravenous hydrocortisone. A marked decline in troponin I and CK levels was observed, indicating resolution of myocardial and skeletal muscle injury. Concurrently, serum Cr and urea normalized, reflecting recovery from acute kidney injury. Mild transient anemia and thrombocytopenia were also noted, both resolving with clinical stabilization. CK = creatine kinase; Cr = creatinine; Hb = hemoglobin; Plt = platelets

Timepoint	Troponin I (ng/L)	CK (U/L)	Cr (mg/dL)	Urea (mg/dL)	Albumin (g/dL)	Hb (g/dL)	Plt (10³/µL)
Reference values	<14	30–200	0.6–1.3	10–50	3.5–5.0	12–16	150–400
Day 1	20,188	2,412	1.64	35.8	3.6	13.3	182
Day 3–5	6,327	2,412	1.9	46	3.6	10.6	80
Day 7–10	1,360	270	1.3	64	2.7	8.7	76
Day 12–13	288	334	1.29	78	2.9	9.8	85

**Figure 3:**
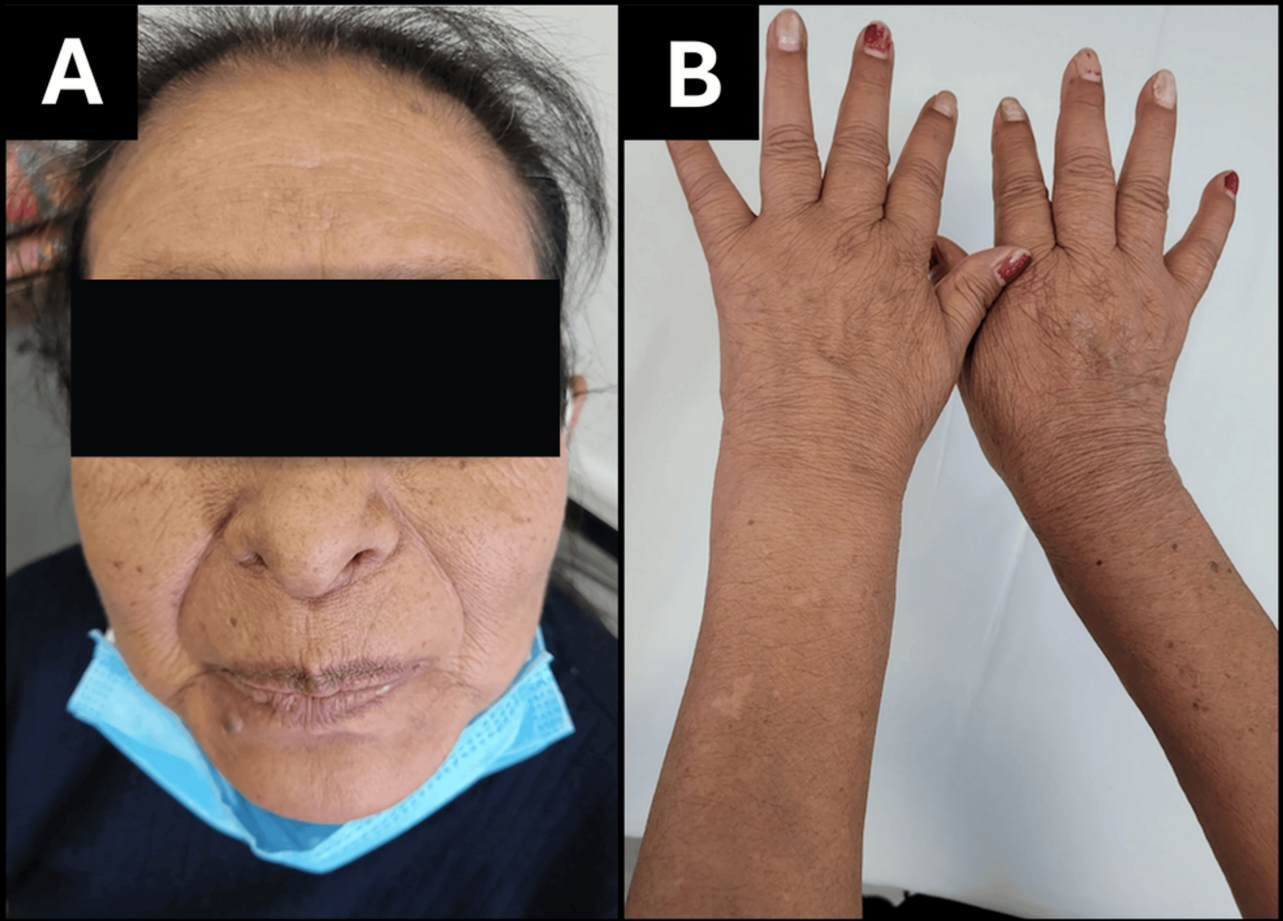
Post-treatment clinical improvement after recovery from myxedema coma. (A) Follow-up photograph showing resolution of facial puffiness, normalization of skin texture, and disappearance of periorbital edema three weeks after discharge. (B) Dorsal view of the hands demonstrating restoration of skin turgor and reduction of xerosis and thickening following thyroid hormone replacement therapy.

The patient was discharged from the intensive care unit with stable hemodynamics, preserved renal function, and complete neurological recovery. At follow-up, the patient was clinically euthyroid under levothyroxine replacement, with full resolution of myopathy, normalization of cardiac function, and no residual deficits (Figure 3).

## Discussion

Myxedema coma is a rare but severe endocrine emergency, with an estimated incidence of 0.22-1 case per million annually [2]. It primarily affects older women, with a female-to-male ratio of 8:1, accounting for nearly 80% of reported cases [1,2]. Our patient, a 66-year-old woman, falls within this epidemiological profile. Thyroid autoantibody testing was not performed at admission due to the emergent presentation; however, the diagnosis of myxedema coma relies primarily on severe biochemical hypothyroidism and characteristic clinical features rather than autoimmune serology. Current guidelines emphasize that markedly elevated TSH with undetectable free T4 is sufficient to establish primary hypothyroidism in the acute setting, and antibody testing does not alter immediate management or diagnostic certainty in decompensated states [2,3].

Classically, myxedema coma is defined as decompensated hypothyroidism with altered mental status and hypothermia, typically accompanied by elevated TSH and a precipitating factor such as infection, trauma, drug exposure, or metabolic stress. However, up to half of reported cases lack an identifiable trigger, as observed in our patient [1-3]. The pathophysiology of multiorgan dysfunction remains incompletely understood. Hypothyroidism-related myopathy has been associated with impaired glycogenolysis, mitochondrial dysfunction, and defective lipid utilization, leading to muscle injury and rhabdomyolysis [7,8]. This mechanism aligns with the markedly elevated creatine kinase in our patient, which normalized following thyroid hormone replacement. Similar enzyme surges during thyroid crisis have been described in untreated Graves’ disease, attributed to circulatory collapse and metabolic failure [8].

Cardiovascular involvement is a key prognostic determinant. Thyroid hormones regulate myocardial contractility, vascular tone, and β-adrenergic sensitivity [1,9,10]. Severe deficiency results in bradycardia, reduced cardiac output, and pericardial effusion [7]. Elevated cardiac biomarkers may reflect impaired clearance and hypothermia rather than true infarction [1,2]. This aligns with our case, in which extreme troponin elevation occurred without electrocardiographic evidence of ischemia. Renal impairment is less common but can arise from hemodynamic and metabolic mechanisms [11]. Reduced cardiac output lowers renal perfusion, while rhabdomyolysis contributes to tubular injury through myoglobin-mediated nephrotoxicity [5,7,9,12]. Seo et al. proposed that impaired glycogenolysis and oxidative metabolism predispose hypothyroid patients to rhabdomyolysis [7]. In our case, renal function rapidly improved after hormone replacement and stabilization of perfusion. Neurological deterioration, ranging from lethargy to coma, represents the hallmark of myxedema coma and may mimic sepsis or other causes of encephalopathy in older adults [4,12,13].

Diagnosing myxedema coma remains challenging due to nonspecific clinical and biochemical features that overlap with sepsis, metabolic encephalopathy, or stroke. Although TSH is typically elevated in primary hypothyroidism, levels may be low or inappropriately normal in secondary (central) hypothyroidism caused by pituitary or hypothalamic dysfunction, which can obscure the diagnosis if free T4 is not measured [2-4]. This distinction is clinically relevant because reliance on TSH alone may delay recognition of decompensated hypothyroidism. In our patient, the markedly elevated TSH with undetectable free T4 supported primary thyroid failure rather than a central etiology. The Popoveniuc scoring system offers a structured approach to overcome this diagnostic ambiguity, integrating clinical and biochemical parameters to support early identification and treatment [3].

Management relies on early recognition and rapid intervention. The American Thyroid Association recommends intravenous levothyroxine as first-line therapy, with liothyronine in selected cases, and hydrocortisone to prevent adrenal crisis [2,3,11,12]. Nevertheless, intravenous formulations are not universally available, and recent evidence supports the efficacy of high-dose oral or enteral levothyroxine under these circumstances. In a single-center series, Rajendran et al. demonstrated that crushed oral levothyroxine administered via a nasogastric tube achieved favorable biochemical and clinical responses, with a survival rate exceeding 90% among patients with myxedema coma [14]. Moreover, classical pharmacokinetic data indicate that levothyroxine absorption occurs predominantly in the duodenum and proximal jejunum, allowing adequate uptake when intestinal perfusion and motility are preserved [15]. In this case, enteral levothyroxine combined with intravenous hydrocortisone led to rapid improvement within 72 hours, including vasopressor withdrawal and successful extubation. Collaboration among endocrinology, neurology, cardiology, and critical care teams enables comprehensive evaluation of hemodynamic, neurological, and endocrine stability while guiding individualized dosing and monitoring strategies. Early interdisciplinary communication is essential to optimize recovery and minimize complications in severe endocrine emergencies such as myxedema coma [1,2,12]. Despite advances in endocrine and critical care management, overall mortality remains between 30% and 60% [1].

## Conclusions

This case underscores that myxedema coma, while uncommon, may present with acute neurological deterioration accompanied by simultaneous multiorgan dysfunction, including myopathy, myocardial injury, and acute kidney impairment. The absence of an identifiable precipitating factor emphasizes the importance of maintaining a high index of suspicion in elderly patients with unexplained encephalopathy and systemic instability. Early recognition, timely initiation of thyroid hormone replacement, and adjunctive corticosteroid therapy were decisive in achieving rapid recovery and full biochemical normalization. Considering the persistently high mortality associated with this condition, continued documentation of atypical presentations remains essential to refine diagnostic awareness and optimize management strategies for this life-threatening endocrine emergency.
